# A novel device in parathyroid autotransplantation for 6 patients with secondary hyperparathyroidism - Case series

**DOI:** 10.1016/j.amsu.2018.09.012

**Published:** 2018-09-19

**Authors:** Xi Wei Zhang, Gang Liu, Xue Feng Tang, Hao Zhang, Jian Ping Huang, Lei Du

**Affiliations:** Department of General Surgery, Shanghai Shu Guang Hospital, China

**Keywords:** Secondary hyperthyroidism, Endoscopic parathyroidectomy, Parathyroid autotransplantation

## Abstract

**Background:**

Secondary hyperparathyroidism(sHPT) is one of the most serious complications in long-term hemodialysis patients. Patients may suffer from metabolic bone diseases, severe atherosclerosis and undesirable cardiovascular events. Endoscopic parathyroidectomy with autotransplantation is a treatment option for those who do not respond to clinical management.

**Methods:**

6 patients with secondary hyperparathyroidism were treated with endoscopic parathyroidectomy and autotransplantation. Pieces of parathyroid tissue were squeezed in our novel self-made device and injected into brachioradialis.

**Results:**

Preoperative symptoms were alleviated, and the serum PTH and alkaline phosphatase levels, hyperphosphatemia, and hypercalcemia were improved or normalized in all 6 patients. The preparation time of parathyroid fragments for autotransplantation was less than 10 min in all 6 patients.Pathological examinations revealed parathyroid cells remains active.

**Conclusion:**

Application of the novel squeezing device is an economic, effective and safe way in endoscopic parathyroidectomy and autotransplantation for patients with secondary hyperparathyroidism.

## Highlights

• A brand-new mincing device for endoscopic parathyroidectomy with autotransplantation.
• A Chinese utility model patent (ZL 2016 2 0280921.2).
• Saving quite considerable preparation time for autotransplantation.

## 1 Introduction

Secondary hyperparathyroidism(sHPT) is a frequent complication of end-stage renal failure, evolving metabolic bone diseases, severe atherosclerosis and undesirable cardiovascular events [1]. Advances in the medical treatment of sHPT have reduced the need for surgery, however 5–10% of patients still require parathyroidectomy [2]. Parathyroidectomy is required for approximately 10%–30% of the patients with more than 10 years of hemodialysis [3]. The best surgical approach for sHPT is still debated and controversy remains. Either subtotal parathyroidectomy or total parathyroidectomy could be performed with or without autotransplantation [4]. Autotransplantation of parathyroid grafts into the sternocleidomastoid muscle have been widely used, however Wells et al. have demonstrated that the forearm muscle can be more advantageous [5]. Subcutaneous injection or transplantation in the forearm was also effective in patients of chronic renal failure [6]. However, parathyroid tissues should be sliced into suitable size for immediate injection and thus we used this device to shorten the preparation time.The stainless steel squeezing device has been designed by Jianping Huang and given a Chinese utility model patent (ZL 2016 2 0280921.2).

## 2 Methods

This research work has been reported in line with the PROCESS criteria [7].

It is a prospective single-centre research for consecutive cases from 2016.Chronic renal failure patients were taking hemodialysis two or three times a week. After 5 years, many patients suffered from metabolic bone diseases, severe atherosclerosis and undesirable cardiovascular events even they were taking calcitriol and a vitamin D analog. And symptoms are getting severer when hemodialysis is going on. Participants of the study were eligible for surgery, and medical treatment for reducing iPTH level was unsatisfactory after 6 months. It is intended to get a 5-year follow-up of these cases even if we only got a 6 months result. According to the inclusion criteria, 6 patients had been performed endoscopic parathyroidectomy and autotransplantation by the same team under general anaesthesia from 2016.

**Fig. 1:**
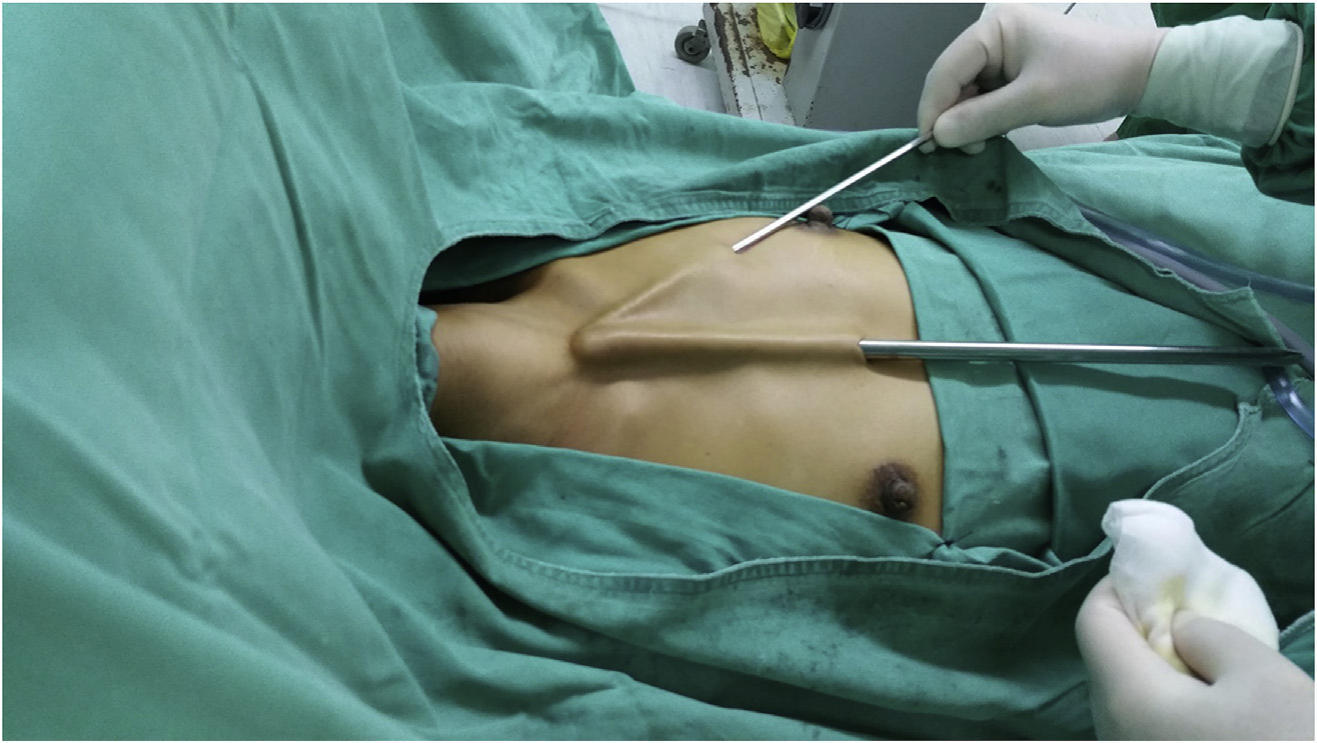
Creating Straight line tunnels.

After the patient was placed in a supine position, a pillow was placed beneath the shoulder to extend the head and neck. A 10-mm incision was made in the intersection point of median thoracic line and bilateral nipples. A metal rod with blunt round end was used to create a straight line tunnel towards sternal notch. Two 5-mm incisions were made in the intersection point of bilateral midclavicular line and the second rib. Also two 5-mm metal rods were used to create straight line tunnels(Fig. 1).

After the blunt dissection, three trocars were inserted through each tunnel. Carbon dioxide gas was injected with a pressure of 8 mmHg.A 30-degree 10-mm endoscope was inserted through the middle 10-mm trocar, a ultrasonic scalpel and a grasping forceps were inserted through two 5-mm trocars. Subplatysmal space was dissected to create a working space from suprasternal fossa to the thyroid cartilage level and laterally to the medial edge of sternocleidomastoid muscles.

**Fig. 2:**
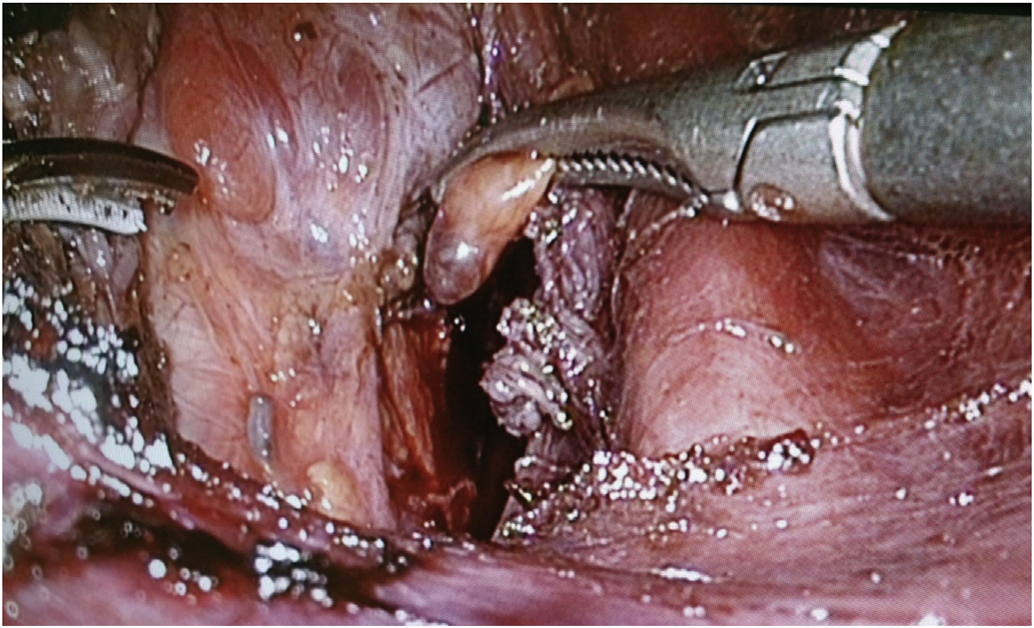
Dissecting parathyroid gland.

A longitudinal incision of the linea alba cervicalis was made, and the bilateral infrahyoid muscle groups were transected to expose the thyroid gland. The posterior surface of the thyroid was explored to find the parathyroid glands according to the surgical anatomy(Fig. 2).

**Fig. 3:**
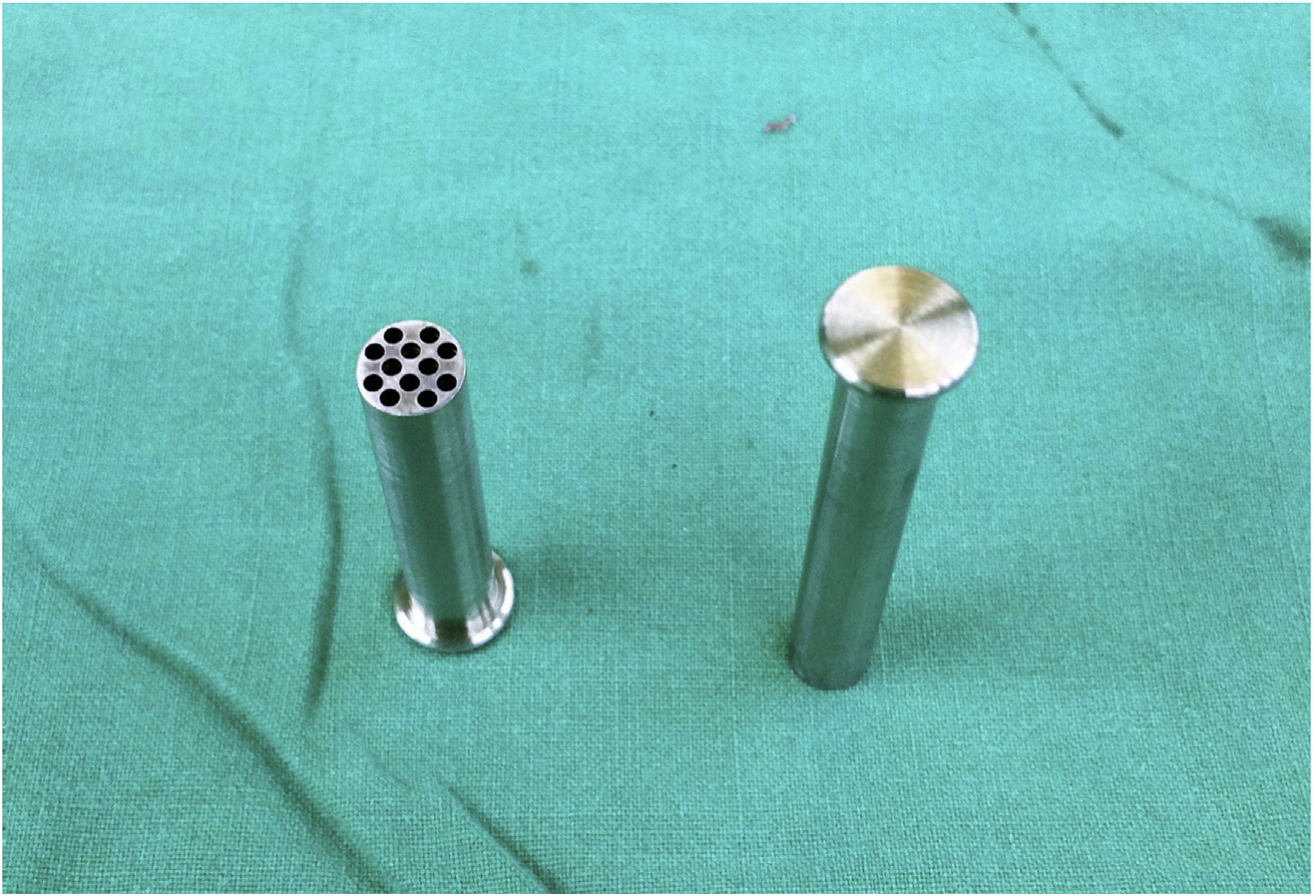
The metal squeezing device.

Removed parathyroid glands were carefully examined in order to select a non-nodular area rich in stromal fat cells for immediate graft implant. The selected parathyroid fragment was gently diced into small pieces measuring approximately 2.0 mm³.The novel device is a metal sleeve which could easily be put into a syringe. The metal sleeve was composed of two parts, the outer part was a hollow tube with multiple holes and the inner part was a solid core(Fig. 3).

**Fig. 4:**
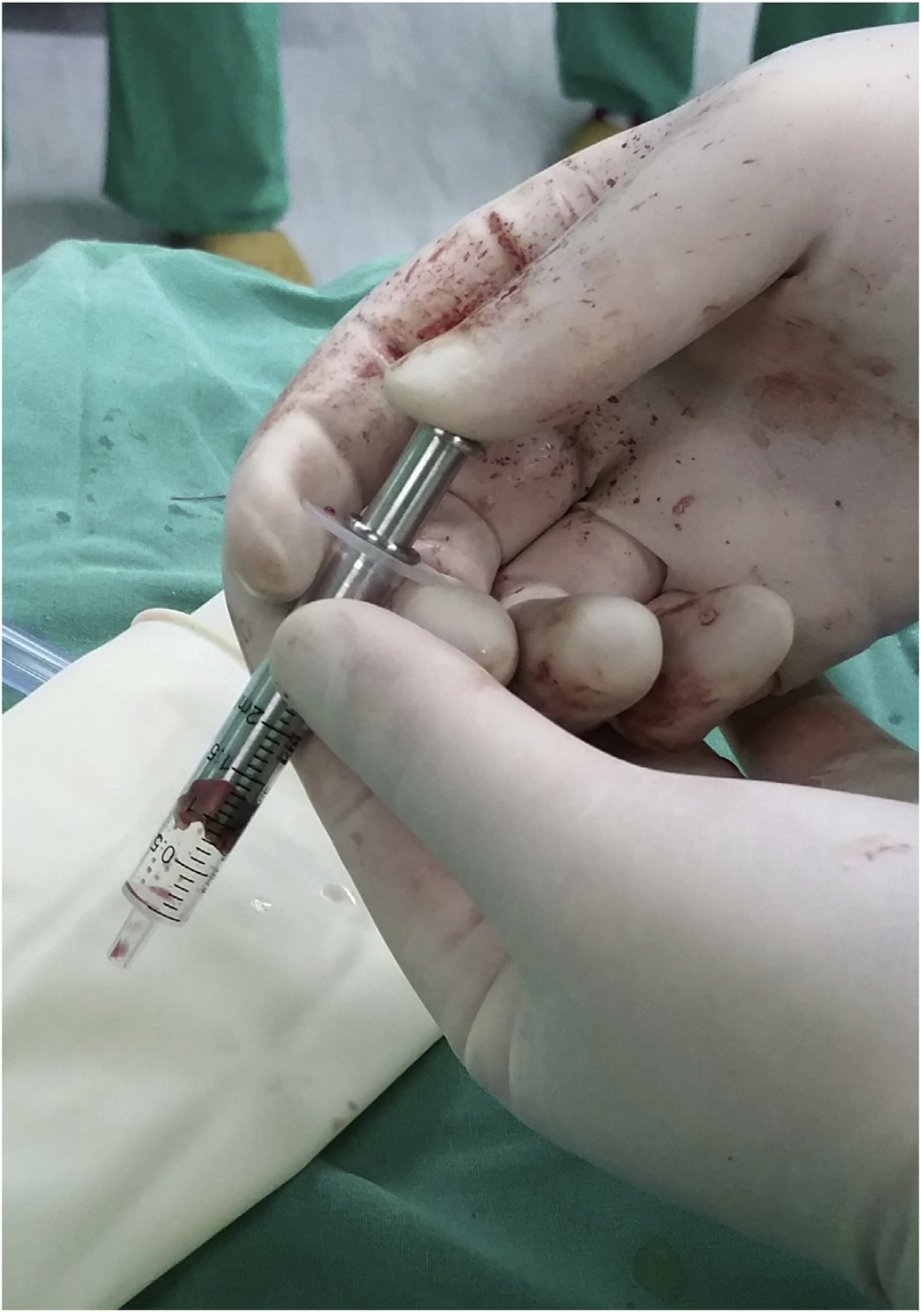
Squeezing parathyroid fragments.

**Fig. 5:**
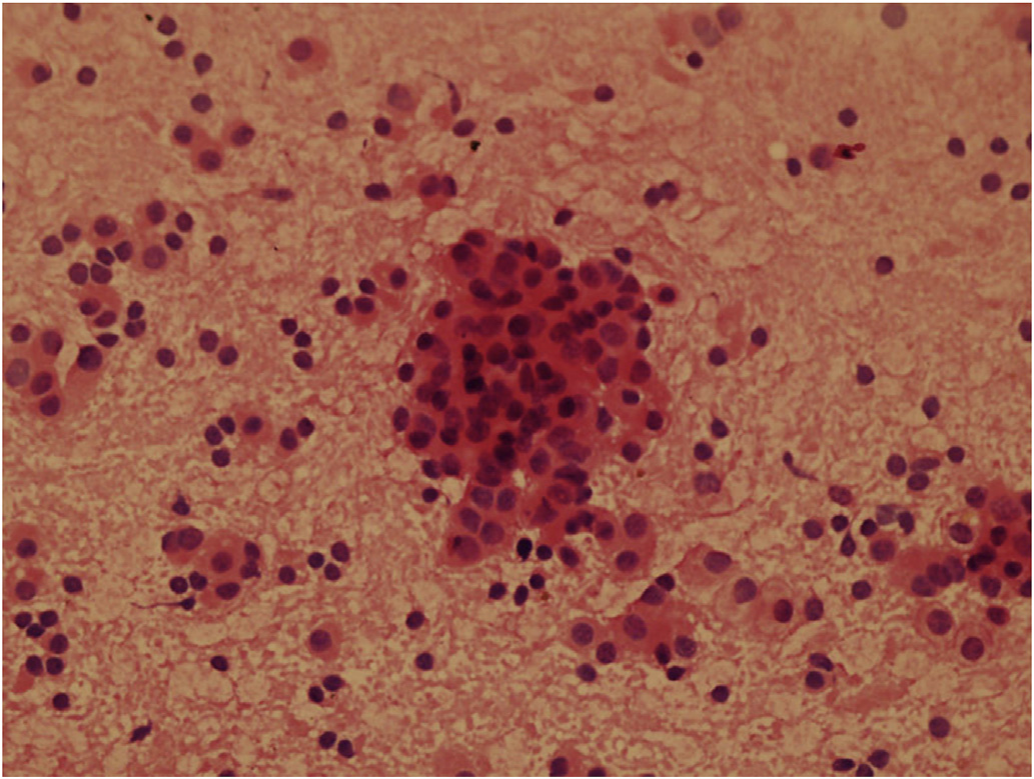
Parathyroid cells after squeezing, HE, × 100.

Parathyroid fragments were roughly diced and put into the metal sleeve, squeezed in a syringe. The parathyroid tissue after squeezing was mixed with normal saline and injected into brachioradialis in the forearm without formation of an arteriovenous (A-V) fistula for hemodialysis (Fig. 4).Compared to implantation in forearm muscles, we spared quite considerable time by using this novel device. This squeezing device has also got a Chinese utility model patent (ZL 2016 2 0280921.2).Pathological examination revealed at least 80% of the parathyroid cells remain active (Fig. 5).

## 3 Results

All six patients were performed endoscopic parathyroidectomy successfully without any complications. No less than four parathyroid glands were removed in 5 patients. However, 3 parathyroid glands were removed in one patient. No laryngeal nerves were apparently injured during the operation, although vocal hoarseness was observed afterward. These symptoms were relieved after conservative treatment. The preparation time of parathyroid fragments for autotransplantation was less than 10 min in all six patients. Using the squeezing device had saved much time while keeping parathyroid tissues active for further injective transplantation. However, more data is needed for comparison and statistics.

The clinical symptoms and biochemical index of sHPT were significantly improved. Pruritus and bone pain was relieved or disappeared within 6 months. By the third postoperative day, the serum calcium level dropped to within or below the normal range in all patients. Recurrence of graft-dependent hyperparathyroidism was not observed in any of the patients during a 6-month follow up and a 5-year outcome was still following up.

## 4 Discussion

Despite of the improvements in medical management, severe sHPT remains a common complication of chronic renal failure. The longer survival of chronic renal disease patients increased the incidence of symptomatic hyperparathyroidism condition [8]. The prevalence of parathyroidectomy in end stage renal disease ranges from 9.2% after 10–15 years of dialysis to 20.8% after 16–20 years of dialysis [9,10]. In patients eligible for surgery, parathyroidectomy is generally considered when hyperparathyroidism is severe and refractory to medical management, usually after a therapeutic trial of calcitriol and a vitamin D analog, or when medical management to reduce iPTH levels results in unacceptable rises in levels of serum calcium and/or phosphorus, or when medical management is not tolerated because of adverse effects [11].

The best surgical approach for secondary hyperparathyroidism remains controversial in the literature. Wells et al., reported their experience in total parathyroidectomy with parathyroid autotransplantation in forearm musculature in 1975.Therefore, total parathyroidectomy with forearm autograft has been considered the standard procedure ever since [12]. However, this technique also has some restrictions. In 1979, Wells reported three (7.5%) patients with graft-dependent hyperparathyroidism, of whom one presented with persistent hyperparathyroidism even after parathyroid graft was excised [13].In order to avoid these undesirable recurrence, other researchers have found alternative receptor areas: Jansson and Tisell, in subcutaneous abdominal adipose tissue [14], Kinnaert et al., in presternal subcutaneous region [15], Monchik et al., in subcutaneous forearm tissue [16], and Lieu et al., into the sternocleidomastoid muscle [17].In our study, we used fresh parathyroid tissue buffered in 2 ml balanced saline in the form of a suspension for intramuscular injection [18]. Pre-transplantation frozen section is sometimes performed if there is doubt on whether the tissue is actually the parathyroid or not, or to reveal whether the tissue is active. However, it is not routinely recommended because of both maintaining maximum parathyroid tissue and avoiding loss of time and cost.

Hyperparathyroidism recurrence and graft-dependent recurrence are both unneglectable after parathyroid autotransplantation. Endoscopic bilateral exploration was performed in all the patients in our study to identify all the parathyroid glands. With the improved visualization and amplification of endoscope, we did not miss any parathyroid glands. Santos [8] reported a graft –dependent recurrence of 9.09% in total 66 patients, and all recurrences occurred in patients under dialysis treatment with no recurrence observed among kidney-grafted patients throughout extensive follow up. In our study, there was no recurrence of hyperplastic parathyroid tissue in the forearm muscles, possibly due to the short follow-up periods. However, we can easily excise it under local anaesthesia or inject anhydrous alcohol to destroy the hyperplastic parathyroid tissue under ultrasonic localization even if it occurs.

The transplanted parathyroid tissue will spontaneously be revascularized and innervated. It begins functioning usually in 2–4 weeks and regains full function in two months [19]. The autograft function may be also indirectly assessed by measuring the level of PTH in serum from the antecubital veins of the grafted and nongrafted arms, with a PTH ratio of 1.5 or greater indicating functional parathyroid tissue [20]. According to the criteria, all of our 6 patients have had satisfactory results.

Since our study was intended as pilot study, limitations were inevitable. First, the number of samples is small and no control group is included. More statistical data is needed to confirm the function of transplanted parathyroid tissue. Second, our follow-up period is only 6 months which may not be long enough to reveal all the complications after surgery. And we also need more experience in dealing with complications as well as prognosis. Third, we do not have other studies with the same surgical approach so as to properly compare surgical results.

## 5 Conclusions

In conclusion, application of the novel squeezing device is an economic, effective and safe way in endoscopic parathyroidectomy and autotransplantation for patients with secondary hyperparathyroidism. It provides ideal outcomes while saving quite considerable time.

## Ethical approval

None.

## Sources of funding

None.

## Author contribution

XiWei Zhang:writing.

Gang Liu:study design.

XueFeng Tang:data collection.

Hao Zhang:data collection.

Lei Du:data analysis.

JianPing Huang:study design and data analysis.

## Conflicts of interest

None.

## Research registration number (UIN)

UIN: researchregistry3254.

## Guarantor

JianPing Huang.
